# Gender-based discrimination shapes psychological safety and professional identity in clinical settings: a qualitative study of nursing, midwifery, and medical students

**DOI:** 10.1186/s12909-026-09965-3

**Published:** 2026-07-30

**Authors:** Tuba Sengul, Seda Sarıköse, Betül Uncu, Ezgi Hasret Kozan Cikirikci, Dilek Yilmaz Akyaz, Nurten Kaya

**Affiliations:** 1 Department of Nursing, Koç University School of Nursing, Koç University Health Sciences Campus, Topkapı, Istanbul, 34010 Türkiye; 2 Department of Midwifery, Faculty of Health Science, Istanbul University-Cerrahpasa, Istanbul, Türkiye; 3 Graduate School of Health Sciences, Koç University, Istanbul, Türkiye

**Keywords:** Gender-based discrimination, Psychological Safety, Professional Identity, Clinical settings, Nursing students, Midwifery students, Medical students, Qualitative study

## Abstract

**Background:**

Gender-based discrimination remains a persistent structural challenge in health professions education and may shape students’ access to learning opportunities, sense of belonging, and professional identity formation. Psychological safety is essential for participation in clinical learning; however, little is known about how gendered experiences are perceived to influence psychological safety, professional identity, and resilience among nursing, midwifery, and medical students.

**Methods:**

This qualitative study explored how nursing, midwifery, and medical students experience gender-based discrimination in educational and clinical learning settings and how they perceive these experiences as relating to psychological safety, professional identity development, and resilience. Guided by critical social theory, the study adopted an IPA-informed interpretative qualitative design. Seven FGDs were conducted with nursing, midwifery, and medical students (*n* = 56). Data were analysed using an iterative, interpretative process supported by MAXQDA. Reporting followed the COREQ guidelines.

**Results:**

Analysis generated three interrelated themes with nine sub-themes. Theme 1, Gendered Professional Identity: Professional Role, Competence, and Status, comprised gender-based labelling of professional roles, gender-coded task allocation framed through “sensitivity versus physical strength,” and glass-ceiling perceptions in career pathways. Theme 2, Inequity in Clinical Learning Opportunities and Psychological Safety, included restricted access to learning, gendered recognition and discrimination, and psychological safety and withdrawal. Theme 3, Resilience Through the Lens of Gender, encompassed competence-based identity construction, supportive academic and institutional ecosystems, and collective voice through professional solidarity and advocacy.

**Conclusions:**

The findings suggest that gender-responsive and psychologically safe clinical learning environments may be important across nursing, midwifery, and medical education. Educators, supervisors, and clinical staff should consider addressing gendered role labelling, inequitable task allocation, and hidden curricula as potential structural barriers to learning and participation. Transparent and auditable learning objectives, gender-responsive supervision, monitoring of task and feedback allocation, and accessible reporting pathways may help reduce perceived inequities and support students’ willingness to speak up. The findings also indicate that moving beyond an exclusive reliance on individual resilience may better support equitable professional identity development, sustainable workforce preparation, and safe patient care. However, these interpretations should be understood within the specific educational, clinical, and sociocultural context of the study.

**Supplementary Information:**

The online version contains supplementary material available at https://doi.org/10.1186/s12909-026-09965-3.

## Introduction

Gender-based discrimination remains a significant and often structurally normalised problem in the educational and clinical training of health professionals [1]. Despite the historical gendering of nursing and midwifery as women-dominated professions and medicine as a men-dominated profession, gendered hierarchies and stereotypes continue to shape educational and clinical settings [1, 2]. These structures may influence students’ access to learning opportunities and clinical experiences, as well as whether they perceive themselves as legitimate members of a professional community [3]. Discrimination is often experienced in subtle, everyday forms; inequities in task allocation and stereotyped expectations regarding competence and communication may reduce participation and willingness to speak up [4, 5]. In hierarchical clinical environments, medical students who identify as women have reported lower psychological safety and greater reluctance to ask questions or voice opinions [4, 6], while nursing and midwifery students may experience professional devaluation and invisibility in interprofessional settings [7, 8].

Research often addresses gender-based discrimination, psychological safety, and professional identity development in fragmented ways, typically within single disciplines and frequently using quantitative designs [3, 4]. Yet clinical education is a core socialisation process through which students learn to define themselves as “nurses,” “midwives,” or “doctors” [9]. Psychological safety, defined as the ability to ask questions, make mistakes, and express uncertainty without fear of negative judgment, is essential for engagement and feedback-seeking [10, 11]. However, gender norms and institutional power asymmetries may undermine psychological safety and contribute to silence or withdrawal [12]. Professional identity development depends on participation, feedback, and belonging and may be adversely affected when students experience or perceive discrimination and feel psychologically unsafe, with potential long-term implications for motivation and professional commitment [13, 14].

Because nursing, midwifery, and medical students share clinical learning environments, teams, and hierarchies, qualitative research examining these groups together and exploring how gender-based discrimination relates to psychological safety and professional identity remains limited. This study, therefore, investigates, from a structural and relational qualitative perspective, how students across these three disciplines experience gender-based discrimination in educational and clinical learning environments and how they perceive these experiences as intersecting with psychological safety and professional identity development.

### Aim

This study examined how gender-based discrimination experienced by nursing, midwifery, and medical students in educational and clinical learning environments shapes their perceptions of psychological safety and influences their professional identity development. In addition, the study qualitatively analysed how students managed these experiences and the individual and collective resilience strategies they developed to protect their psychological safety and professional identities.

### Research questions

This study sought to answer the following research questions:

1. How did nursing, midwifery, and medical students experience gender-based discrimination in educational and clinical learning settings?
2. How did these experiences affect students’ perceptions of psychological safety?
3. How did gender-based discrimination influence students’ professional identity development processes?
4. What resilience strategies did students develop and implement to manage and cope with these experiences?

## Methods

### Study design

This study adopted an IPA-informed interpretative qualitative design, informed by critical social theory, to examine how nursing, midwifery, and medical students experience and interpret gender-based discrimination in educational and clinical learning environments. Rather than applying IPA as a strictly idiographic, interview-based method, we used IPA principles to guide an interpretative analysis of meaning-making, lived experience, and sense-making within focus group discussions. This approach was appropriate because the study aimed to understand both individual accounts of gender-based discrimination and the interactional ways in which these experiences were confirmed, contested, or elaborated within peer groups. The critical social theory lens further supported interpretation of how students’ accounts were situated within broader social, cultural, and institutional power relations. The study was conducted and reported in accordance with COREQ [15] (see Additional File 1).

### Theoretical framework

This study applied an integrated framework combining Gendered Organisational Culture, Psychological Safety, and Professional Identity Formation to examine how gender dynamics in educational and clinical learning environments shape learning, belonging, and identity development among nursing, midwifery, and medical students. Acker’s theory located discrimination within historically gendered institutional systems and power relations, highlighting organisational practices that regulate participation, voice, and access to learning and recognition [16, 17]. Psychological safety guided interpretation of students’ willingness to ask questions, express uncertainty, and take interpersonal risks without fear of negative consequences, including gendered patterns of silence and visibility in hierarchical contexts [10]. Professional identity formation framed identity as developing through participation, recognition, and belonging, enabling analysis of how gendered experiences shaped students’ perceived legitimacy and how reduced psychological safety could disrupt identity development and prompt coping strategies [18, 19]. Together, these lenses supported a multi-level interpretation of structural conditions, relational effects, and the developing professional self.

### Participants and setting

Data were collected between 11 December 2025 and 15 January 2026 from undergraduate nursing, midwifery, and medical students at one foundation and two public universities in Türkiye. Eligible participants had completed at least one clinical placement. Using purposive maximum variation sampling, we sought diversity in gender identity and year of study to support an interpretative exploration of meaning-making across varied clinical learning experiences. Sample adequacy was guided by information power principles [20], considering the specificity of the study aim, the relevance of participants’ clinical placement experiences, the use of an integrated theoretical framework, the quality of dialogue within focus groups, and the cross-group analytic strategy. Data adequacy was assessed iteratively during data collection through field notes, post-focus group debriefings, and preliminary analytic memos. By the final focus groups, the main experiential and meaning-making patterns had been sufficiently developed across nursing, midwifery, and medical students; later discussions mainly elaborated, confirmed, and nuanced these patterns rather than altering the overall interpretative account. Therefore, additional data collection was considered unlikely to substantially alter the overall interpretation, and recruitment was ended with 56 participants. Overall, 56 students participated in seven focus group discussions (discipline-specific and mixed), with approximately 7–10 participants per group; recruitment was voluntary.

#### Inclusion criteria

- Enrolment in an undergraduate nursing, midwifery, or medical programme in Türkiye.
- Completion of at least one clinical placement or clinical rotation within the relevant programme.

#### Exclusion criteria

- Being under 18 years of age.
- Having no clinical placement experience.

### Data collection

Eligible students were recruited purposively through the researchers’ academic and professional channels and personal networks. After receiving an information sheet, students who agreed to participate were emailed a Zoom link; sessions were scheduled according to availability, and online informed consent was obtained. FGDs were selected rather than individual interviews because the study aimed to explore not only individual experiences but also the shared and socially negotiated meanings of gender-based discrimination within clinical learning environments. The group format enabled participants to reflect on, compare, and respond to one another’s accounts, thereby providing insight into how gendered norms, professional identity, and psychological safety were constructed and negotiated through interaction.

Data were generated through discipline-specific focus groups and one mixed interprofessional focus group, and participants completed a brief demographic form before each session. A semi-structured guide comprising 15 framework-aligned questions and follow-up prompts (Table 1) was used and piloted with one student from each discipline. Pilot data were excluded from the analysis and used only to inform minor revisions to the guide. Focus groups were conducted via Zoom by three doctoral-trained qualitative researchers who identified as women, lasted 60–90 min, and were audio-recorded. Field notes, post-session debriefings, and analytic memos were used to document group dynamics and preliminary interpretations. Data adequacy and the recurrence and elaboration of experiential meaning-making patterns were considered throughout data collection. An IPA-informed approach was used to examine meaning-making as it emerged through both individual accounts and group interaction [21].

**Table 1:** Semi-structured questions

Introduction	• Do you think gendered expectations influence students’ treatment in educational and clinical settings (task allocation, assessment, feedback)?
Transition	• Can you share an instance where you felt advantaged or disadvantaged because of your gender, and how you interpreted it?
Key Questions	• How do you think leadership, competence, and authority are perceived across genders in your context? • Are there implicit gendered norms or “invisible rules” in your programme or clinical placements? How do they shape learning and participation? • Have you observed the same behaviour (assertiveness, silence, making mistakes) being judged differently depending on gender? • Have you encountered implicit messages that certain clinical tasks are “women’s work” or “men’s work”? • In learning or clinical environments, how safe do you feel asking questions, disagreeing, or admitting uncertainty? What factors shape this? • Do you think the interpersonal or professional consequences of speaking up—or acknowledging mistakes vary by gender? • When do you most feel like a “real” nurse/midwife/doctor? Does gender affect that sense of legitimacy? • How have unfair, disrespectful, or discriminatory experiences influenced your professional identity and confidence? • Have you experienced gender-related exclusion or discrimination, and what helped you cope or recover? • What forms of support (mentors, role models, peers) have strengthened your sense of belonging and professional development? • Have you ever felt pressured to change your behaviour (be quieter, more compliant, more assertive) because of gender norms? How has this affected you? • Do societal expectations about “appropriate” genders for certain health professions affect your sense of belonging or career plans? • How do you navigate tensions between “being yourself” and “fitting in” in the profession? • What needs to change to make educational and clinical environments more equitable, inclusive, and psychologically safe? • If you could give one piece of advice to a student starting tomorrow, what would it be?
Follow-up Questions	• Could you tell me more about that? • Could you give me an example?
Closing	• Is there anything else you would like to add?

### Data analysis

Data were analysed using an IPA-informed interpretative approach [22]. FGDs were treated as compatible with this approach because meanings were understood as both individually experienced and interactionally shaped through agreement, contrast, elaboration, silence, and emotional emphasis within the group setting [21, 22]. To balance attention to group dynamics with individual lived experience, analysis proceeded in three stages. First, transcripts were read iteratively to identify individual experiential claims, attending to participants’ language, metaphors, emotional emphasis, pauses, and personal accounts of meaning-making. Reflexive notes captured initial impressions and analytic questions, making visible how researcher positioning could shape interpretation [22].

Second, each focus group was analysed as an interactional context, with attention to how participants affirmed, challenged, extended, or qualified one another’s accounts. Discursive dominance, silences, and moments of collective recognition were documented through analytic memos. Third, experiential and interpretative patterns were developed within each focus group and then compared across discipline-specific and mixed groups to identify convergences, divergences, and tensions across nursing, midwifery, and medical students [21, 22]. Consistent with IPA’s double hermeneutic, the researchers interpreted participants’ interpretations of their experiences while remaining attentive to the influence of group interaction on meaning-making [22]. The analysis therefore preserved attention to individual lived experience while also recognising that gendered professional identity, psychological safety, and discrimination are socially negotiated within clinical learning cultures. Finally, themes were linked interpretively to the integrated theoretical framework, using gendered organisational culture, psychological safety, and professional identity formation as analytic lenses rather than pre-set categories, and combining inductive with interpretive–abductive reasoning [23]. MAXQDA-24 supported coding, organisation of interpretative patterns, and audit trails. Participants were assigned anonymised codes indicating the programme and the sequence of focus groups (N3, G1; MD2, G2; M4, G5).

### Researcher positionality and reflexivity

The research team consisted of doctoral-trained researchers who identified as women and had academic and professional experience in health professions education, nursing, midwifery, qualitative research, and gender-related issues in educational and clinical settings. These backgrounds provided contextual sensitivity to the clinical learning environments under study but also required ongoing reflexivity regarding how professional experience, the researchers’ own gender identities and assumptions, and familiarity with health professions education could shape data generation and interpretation. To address this, the researchers engaged in reflexive memo-writing, post-focus group debriefings, and team-based analytic discussions throughout the study. During analysis, attention was given to both shared and divergent accounts across nursing, midwifery, and medical students, and interpretations were repeatedly checked against participants’ narratives rather than imposed solely on the basis of the researchers’ prior assumptions. This reflexive process was intended to enhance transparency regarding how meanings were interpreted and how the integrated theoretical framework was applied.

### Rigour and trustworthiness

The methodological rigour of this study was ensured in accordance with Lincoln and Guba’s [24] criteria for qualitative research: credibility, dependability, confirmability, and transferability. Credibility was supported through the generation of rich, contextually grounded data during the FGDs, cross-group comparisons, and ongoing analytic deliberations. The researchers’ assumptions, positionalities, and potential influences on the analytic process were continually interrogated through reflexive notes and team-based analytic discussions. Dependability and confirmability were strengthened by maintaining detailed documentation of analytic decisions, reflexive records, and the stages through which experiential and interpretative patterns were developed. Transferability was supported through a thick description of the research context, participant characteristics, and the learning environments.

### Ethical considerations

Ethical approval was obtained from the Koç University Committee on Human Research (Ethics Committee No and Date: 2025.560.IRB3.224, 04.12.2025). The study was conducted in accordance with the Declaration of Helsinki and ethical principles for qualitative research [25].

## Results

### Participant characteristics

FGDs included 56 students across seven sessions (midwifery: 2; nursing: 3; medicine: 1; mixed: 1). The mean age was 21.98 ± 1.56 years (range: 19–26). Regarding gender identity, most participants identified as women (*n* = 46, 82.1%), followed by those who identified as men (*n* = 8, 14.3%); one participant identified as non-binary (*n* = 1, 1.8%), and one preferred not to disclose their gender identity (*n* = 1, 1.8%). Most participants were enrolled at a foundation university (*n* = 35, 62.5%). The sample comprised 28 nursing students (50.0%), 16 midwifery students (28.6%), and 12 medical students (21.4%). By year of study, 19 participants (33.9%) were in Year 2, 13 (23.2%) in Year 3, 14 (25.0%) in Year 4, and five each in Years 5 and 6 (both 8.9%). Fewer than half reported having received prior education on gender, equality, or diversity (*n* = 22, 39.3%), whereas 34 (60.7%) reported that they had not received such education. Twelve students (21.4%) identified as belonging to a minority group within their programme or clinical setting, 41 (73.2%) did not, and three (5.4%) preferred not to answer. More than half (*n* = 33, 58.9%) reported having experienced or witnessed gender-based discrimination in educational settings, whereas 23 (41.1%) reported no such experience (Table 2).

**Table 2:** Demographic characteristics of students (*N* = 56)

Variables	Mean ± SD	Min.-Max.
*Age*	21.98 ± 1.56	19–26
*Gender identity*	**n**	**%**
Woman	46	82.1
Man	8	14.3
Non-binary	1	1.8
Prefer not to disclose	1	1.8
*Academic department*		
Nursing	28	50.0
Midwifery	16	28.6
Medicine	12	21.4
*University Type*		
Public	21	37.5
Foundation	35	62.5
*Class Year*		
2	19	33.9
3	13	23.2
4	14	25.0
5	5	8.9
6	5	8.9
*Receiving education on gender*,* equality*,* and diversity*		
Yes	22	39.3
No	34	60.7
*Minority status in your programme/clinical setting*		
Yes	12	21.4
No	41	73.2
Prefer not to say	3	5.4
*Experiencing or witnessing gender discrimination in educational settings*		
Yes	33	58.9
No	23	41.1

### Themes and sub-themes

Following the IPA-informed interpretative analysis of the FGDs, participants’ narratives were organised into three overarching themes and nine sub-themes (Table 3).

**Table 3:** Mapping of Themes and Sub-themes to the Integrated Theoretical Framework

Themes	Sub-themes	Related Theory and Key Concepts
Gendered Professional Identity: Professional Role, Competence, and Status	· Gender-Based Labelling of Professional Roles	Professional Identity Formation: recognition, professional belonging, role legitimacy, and identity misrecognition. Gendered Organizational Culture: occupational gender coding and gendered role expectations.
	· Sensitivity or Physical Strength? Defining Professional Roles Through Gendered Codes	Gendered Organizational Culture: gendered division of labor, task allocation, and competence stereotypes. Professional Identity Formation: perceived fit between gender, role expectations, and professional competence.
	· The Glass Ceiling and Career Pathways	Gendered Organizational Culture: status hierarchy, leadership norms, career progression barriers, and gendered authority. Professional Identity Formation: anticipated career legitimacy and professional self-positioning.
Inequity in Clinical Learning Opportunities and Psychological Safety	· “Don’t Come, Wait”: Gender-Based Access to Clinical Learning	Gendered Organizational Culture: gendered access to practice, exclusionary norms, and unequal learning opportunities. Psychological Safety: participation safety and perceived risk in accessing clinical learning.
	· Gendered Recognition and Discrimination	Professional Identity Formation: recognition, professional legitimacy, belonging, and perceived value. Psychological Safety: confidence, visibility, and willingness to participate.
	· Psychological Safety and Withdrawal	Psychological Safety: speaking up, question-asking, interpersonal risk, silence, and withdrawal. Gendered Organizational Culture: gendered power relations and normalization of gender-biased discourse.
Resilience Through the Lens of Gender	· “Self-Improve, Break the Norms”: Building Professional Identity Through Competence	Professional Identity Formation: competence development, self-efficacy, professional legitimacy, and perceived fit. Critical social theory lens : individualized resilience and the risk of shifting structural responsibility onto students.
	· A Supportive Ecosystem: Academic Mentorship and an Empowering Institutional Culture	Psychological Safety: supportive supervision, mentorship, reporting safety, and institutional trust. Professional Identity Formation: role modeling, belonging, and identity affirmation.
	· Collective Voice: Professional Solidarity and Advocacy	Psychological Safety: speaking up, peer support, and protection against interpersonal risk. Critical social theory lens: collective advocacy, resistance to normalized inequality, and challenging gendered hierarchies.

#### Theme 1: gendered professional identity: professional role, competence, and status

Participants described professional identity in educational and clinical settings as being influenced by gendered role expectations, competence judgements, and status hierarchies. Findings were interpreted through the lenses of gendered organisational culture [16] and professional identity formation [18, 19]. Three sub-themes were identified: (i) gender-based labelling of professional roles, including the misattribution of professional titles and the gendered association of nursing, midwifery, and medicine with particular identities and attributes; (ii) gender-coded definitions of professional roles and task allocation, often organised through a “sensitivity versus physical strength” dichotomy and assumptions about “women’s work” and “men’s work”; and (iii) perceived glass-ceiling barriers and gendered inequalities in career progression, leadership opportunities, and positions of authority. Overall, participants’ narratives suggested that professional recognition, role allocation, task-sharing, clinical credibility, and career advancement could be negotiated and organised along gendered lines.

##### Sub-theme 1.1 gender-based labelling of professional roles

Participants reported that professional identity is frequently labelled through gendered associations, which may contribute to the misattribution of professional titles and roles and to evaluations of professions being filtered through gendered perceptions. They noted that the entrenched schema of “doctor = man; nurse and midwife = woman” in clinical settings may influence how individuals’ professional identities are initially perceived. In participants’ accounts, this was illustrated through examples such as doctors who identified as women being assumed to be nurses: *“Sometimes*,* during clinical placements*,* when patients and relatives see that I am a woman*,* they immediately call out*,* ‘Nurse*,* could you take a look?’ Even when I say I am a doctor*,* some still insist on calling me ‘nurse*,*’ as if women cannot be doctors… That’s the perception…” (MD4*,* G4).* Similarly, participants highlighted that men in nursing may be perceived as doctors and that this perception reflects patients’ gender–occupation matching: *“During clinical placements*,* patients can think we are doctors. When I say I’m a nurse*,* they don’t know how to address me. Some say ‘man nurse.’ In patients’ minds*,* doctors are men… women are more likely to be nurses…” (N3*,* G3).*

Participants emphasised that this ambiguity in role attribution is not limited to clinical encounters. In particular, they suggested that the presence of men in nursing is still not fully “established” in society, which may result in men in nursing being attributed alternative professional roles. One participant elaborated: *“Unfortunately*,* men in nursing are not yet fully accepted in our society… People either see us as ‘doctors’ or place us in a ‘caregiver’ position… When they hear ‘nurse*,*’ they mostly think of a woman.” (N2*,* G5).* In addition, participants noted that nursing and midwifery are sometimes defined through attributes culturally associated with women, such as “motherhood” and “compassion,” and that men may therefore be perceived as socially “incongruent” with nursing and midwifery roles. As one participant described: *“Patients may imagine nurses as loving and compassionate*,* like a mother; so for men*,* being a doctor*,* which implies strength*,* may be perceived as more fitting*,* whereas for women*,* nursing and midwifery seem compatible with the role of a compassionate mother…” (N5*,* G6).* Participants also reported that framings of nursing and midwifery as “women’s professions” may be accompanied by judgements about professional prestige. One participant illustrated this through everyday discourse: *“Even my relatives*,* when I said I would become a nurse*,* responded dismissively: ‘A man should be a doctor; this is a woman’s profession.’ They say things like that.” (N3*,* G3).*

Participants further expressed divergent views regarding men’s presence in nursing and midwifery. Some narratives linked men’s participation to increased visibility and prestige for the profession, whereas others suggested that the status ascribed to men could also generate concerns about women’s positioning within the profession. One participant reflected: “*At present in our country*,* unfortunately*,* there are no men working as midwives*,* even though there are many men working as obstetricians… I think men’s presence in midwifery would positively affect our professional status because*,* in patriarchal societies*,* men are associated with power and status…” (M4*,* G1).*

##### Sub-theme 1.2 sensitivity or physical strength? defining professional roles through gendered codes

Participants described the allocation of professional roles and task-sharing as being gendered through a dichotomy between “sensitive skills/fine work” and “physical strength/heavy work.” One participant noted that women were often positioned in tasks requiring detail, care, and follow-up, whereas support from men could be expected for tasks requiring physical exertion or crisis management: *“Women can approach wound care more meticulously and in a more organised way*,* but if a patient needs to be turned*,* help from a colleague who is a man is always expected…” (N7*,* G5).* In contrast, some participants argued that “heavy work” alone was insufficient as a basis for inferring professional role suitability. One participant problematised this rationale, emphasising how the societal assignment of professions could be tied to gender whenever physically demanding roles were invoked: *“When it comes to heavy work and roles in clinics*,* it’s as if society determines people’s professions according to their gender. Orthopaedics is ‘heavy work’—so what would a woman doctor be doing there? ‘You can’t be a surgeon; it’s heavy work*,* you will be a mother; surgeons can’t be mothers…’ I can’t say that ‘only men should be surgeons’ just by taking heavy work as the basis.” (MD3*,* G7).*

Participants also referred to implicit yet palpable boundaries between “women’s work” and “men’s work” in clinical settings, boundaries that may not be openly articulated but are experienced in practice. This was captured in the following account: *“In the clinic*,* there are actually points where we say ‘women’s work’ and ‘men’s work.’ There are some task definitions with invisible boundaries that are not stated explicitly.” (M1*,* G2).* In addition, some participants noted that assumptions about “strength” positioned men as more suitable for certain tasks: *“Take mobilisation*,* for example; generally*,* men are stronger… men can do it more easily.” (M4*,* G2).* Another participant similarly observed: “Unfortunately, there is a perception that orthopaedic surgery requires strength and that surgeons who are men do it better.” (MD1, G4).

Conversely, other participants highlighted that within this gendered division of labour, the workload often remained with women, creating visible tensions within teams. One participant reflected: *“When I started placements*,* I saw that in our clinical environment*,* it’s as if there is no job suitable for men; everyone acts that way. Our family structure is also like that; women somehow work more*,* as if everything is supposed to be done by women.” (M3*,* G1).* Another participant described a similar dynamic: *“There was a man who was a nurse on the ward… but he stayed inside. From his perspective*,* care practices were women’s work. That day*,* there was also a seriously ill patient*,* and the women nurses and we students helped the patient… the men were inside chatting.” (N6*,* G6).*

Finally, some participants noted that gender-based care preferences could lead to practical rearrangements in clinical settings. For example, one participant described a situation in which a man receiving care did not want a staff member who was a woman to perform a procedure such as catheterisation: *“We had an experience where we called a nurse who was a man from another clinic… and this is something that disrupts the clinic and the workflow.” (N5*,* G6).*

##### Sub-theme 1.3 the glass ceiling and career pathways

Participants emphasised that men may advance more rapidly along career pathways and that trust in men can be higher at critical decision points, such as appointments to managerial roles. This was articulated in accounts such as: *“Men climb the career ladder faster; this is true in both nursing and medicine… In our society*,* trust in women is lower in every respect. People think men in nursing are more highly valued*,* and they assume that a woman doctor cannot be trusted.” (N3*,* G3).* Common explanations offered to legitimise the attribution of responsibility and leadership to men were reflected in statements such as: *“I’ve heard so many things like*,* ‘In this hospital*,* among nurses too*,* they immediately make men team leaders’… they see them as more forward-thinking*,* more proactive*,* and faster at managing crises…” (N2*,* G7).*

Alongside these perceived patterns of advancement, participants highlighted the lower visibility of women in senior and managerial positions, as well as the everyday stereotypes that sustain this imbalance. One participant described the persistence of derogatory assumptions about women’s leadership capacity: *“Because people still think ‘women have long hair and short minds*,*’ I don’t think we see many women managers in midwifery*,* nursing*,* or medicine; even chief physicians are always men.” (M3*,* G1).* Another participant questioned how managerial roles are culturally associated with men, highlighting assumptions related to caregiving responsibilities: *“If a managerial position opens if the applicant is a woman*,* people think she will go on maternity leave*,* she will have to take care of her child*,* so ‘let’s not choose her’; but if the applicant is a man*,* then ‘let’s choose him’?” (N4*,* G7).*

#### Theme 2: inequity in clinical learning opportunities and psychological safety

Across the focus groups, participants reflected on whether clinical learning opportunities, participation in practice, learning through observation and hands-on experience, professional recognition, and psychological safety were experienced in gender-equitable ways. They reported that access to learning could be directly restricted in some contexts, whereas in others, opportunities and recognition appeared to diverge through gendered patterns of value attribution. Participants also described how judgemental attitudes, stereotypical expectations, and gendered interactions could reduce their willingness to ask questions, express opinions, or speak up, sometimes contributing to hesitation or withdrawal. This theme was interpreted through the lenses of gendered organisational culture [16] and psychological safety [10].

##### Sub-theme 2.1 “Don’t Come, Wait”: gender-based access to clinical learning

Participants reported that, in some clinical settings, students could be excluded from specific procedures through directives such as “don’t come” or “wait,” effectively positioning them outside the learning process: *“During my summer placement*,* because I was a nursing student who was a man in the obstetrics and gynaecology clinic*,* when procedures were being performed in some rooms*,* they said*,* ‘Don’t come*,* wait.’” (N3*,* G3).* In certain contexts, this exclusion was described as creating learning opportunities that functioned as an “advantage” for one group and a “disadvantage” for another. One participant explained: *“While I was working in the non-stress test (NST) room*,* a pregnant patient did not want nursing students who were men to come in. That day*,* I performed countless NSTs… My friends who were men were left sitting in the corridor and became bored. For me*,* it became an advantage…” (N5*,* G6).*

When interpreting these experiences, participants noted that gender-based preferences, particularly in care-related procedures, were often justified in terms of privacy, modesty, and the right to choose. They argued that, as a result of patients’ preferences, nursing and midwifery students in particular could experience unequal access to clinical training opportunities. This rationale was illustrated in the following account: *“Because the patient is the same gender*,* they want someone of the same gender to touch them; maybe they want only someone of their own gender to see their private areas. It might be because of embarrassment… But*,* for example*,* they can’t do this to intern doctors; intern doctors can receive training comfortably in obstetrics placements and perform procedures.” (M2*,* G1).* Conversely, some narratives indicated that preferences could also operate in the opposite direction. One participant provided the following example: *“Even though I state that I am a doctor*,* especially in interventional procedures*,* patients don’t trust us and say*,* ‘Isn’t there a physician who is a man—let him insert my drain.’” (MD3*,* G4).*

##### Sub-theme 2.2 gendered recognition and discrimination

Participants reported that, even when similar levels of performance and the same skills were demonstrated in clinical and practice settings, recognition could differ by gender. During the discussions, nursing and midwifery students in particular described students who identified as men as being framed as “rare” and therefore rewarded more visibly, whereas students who identified as women could be positioned as more “ordinary” despite performing the same work. This differential recognition was articulated explicitly in one participant’s account: *“During clinical placements*,* especially educators or mentor nurses can approach students who are men in a more motivating way… with applause: ‘Bravo*,* you did it*,* well done*,* congratulations.’” (N6*,* G6).*

Another participant noted that women’s work could be framed as “already expected,” thereby shaping the level of appreciation: *“For example*,* I submit an excellent care plan to my instructor*,* and I expect acknowledgement*,* but it doesn’t come. We have become the ‘usual’ ones. ‘Okay*,* of course you will do it*,* you are a woman; this is your job…’” (M2*,* G1).* Participants described this divergence as an inequity that could negatively shape their emotional responses and perceptions of the learning experience. One participant summarised this perception as follows: *“Even though we do the same work*,* I think they receive more appreciation. In nursing*,* I definitely think instructors who are women protect and support students who are men more… Even regarding absenteeism or being late*,* they are more tolerant toward students who are men…” (N6*,* G7).*

Some participants, however, suggested that institutional culture and educators’ approaches might not produce such differentiation in all contexts. In particular, some medical students reported experiencing a more egalitarian approach during training and practice, noting that recognition and feedback did not diverge by gender. Within these narratives, participants emphasised that instructors “treated everyone equally” and that no gender-based differences were experienced in assessment processes. As one participant stated: *“…I definitely did not see such discrimination. It may be related to the culture of our university*,* because sometimes I hear from friends studying medicine elsewhere that some instructors say women students are more meticulous and organised and therefore assign patient follow-up to them during intensive care placements…” (MD5*,* G7).*

##### Sub-theme 2.3 psychological safety and withdrawal

Participants reported that, in learning environments such as classrooms, laboratories, clinical settings, and ward rounds, the willingness to ask questions, express opinions, and speak up, as well as tendencies to hold back or withdraw, could vary by gender in relation to prevailing gender perceptions. One participant indicated that, in clinical settings, asking questions of certain individuals did not feel easy: *“In the clinic*,* I honestly can’t ask patients who are men many questions. They seem very judgemental to me.” (N2*,* G3).*

In addition, some participants noted that “judgemental attitudes” in certain courses reduced their willingness to speak and that this was experienced as a form of hesitation shaped by “confidence-undermining” experiences: *“I don’t know*,* maybe things like the perception of women in our society and the value given to women play a role*,* but in some classes I can’t comment comfortably… I hesitate because of judgemental or confidence-breaking things.” (N6*,* G6).* Participants further emphasised that speaking up and voicing opinions during education was not solely a matter of personal inclination; rather, social perceptions could contribute to gendered dynamics that rendered men more visible. In this regard, one participant stated: *“I think people who are more confident and more assertive are more visible*,* and these are often students who are men*,* and they are listened to more by instructors.” (N5*,* G4).*

Another dimension of this sub-theme was that disparaging or undermining societal remarks could render learners’ identities fragile and reduce their professional motivation. Participants illustrated the perceived impact of stereotypical statements directed at both women and men through examples such as: *“I love my profession*,* but patients’ thoughts reduce my motivation. One of my patients said*,* ‘How can a man be a nurse? If only you had studied a bit more and become a doctor.’” (N3*,* G3). Another participant similarly reflected: “I want to choose orthopaedics for residency*,* but society’s view is very clear: ‘Women can’t be good surgeons; I can’t trust them.’ It’s exhausting…” (MD3*,* G4).*

Finally, participants noted that perceptions of psychological safety in clinical contexts were not limited to intra-team relationships; interactions with patients’ relatives were also perceived as influencing these experiences. *One participant described a gendered sense of risk: “I think a patient’s relative can be much harsher toward a doctor or nurse who is a woman; I observe that they behave more disrespectfully.” (N2*,* G4).* Another participant added: *“There are even incidents of violence*,* and the idea that violence can be directed at nurses or midwives is more widespread; because ours are women-dominated professions*,* this is unfortunately related to the value given to women.” (M5*,* G2).*

#### Theme 3: resilience through the lens of gender

Participants described resilience in response to gender-related devaluation and exclusion in clinical and educational settings as involving both individual resources, such as self-improvement and self-efficacy, and collective and institutional resources, including solidarity, advocacy, mentorship, role models, and supportive organisational cultures. Their accounts suggested that resilience was experienced not only as an individual capacity but also as a relational and professionally supported process. This theme was interpreted through the frameworks of professional identity formation [18, 19] and psychological safety [10].

##### Sub-theme 3.1 “Self-Improve, Break the Norms”: building professional identity through competence

Participants emphasised that developing skills and demonstrating competence could help them respond to gender-based prejudices. In particular, students offered explicit recommendations for challenging gendered biases, using expressions such as “self-improve,” “strengthen your practical skills,” and “break the norms.” In this regard, one participant stated: *“I think we are the ones who will raise awareness in society by standing firm against everyone who says ‘this is women’s work’ or ‘this is men’s work.’ For that*,* we first need to develop ourselves professionally so that we are respected*,* and of course*,* at this stage*,* we also need to stay psychologically strong.” (M1*,* G7).*

Within the same sub-theme, some participants explicitly stated that professional competence is not determined by gender but rather by individual characteristics and skills. One participant articulated this as follows: *“I can say that professions do not have a gender. I think it is less about gender and more about the person. For example*,* a woman can be a very good doctor*,* and a man can be a very good nurse. A man might even provide better care than a woman and approach patients more compassionately; we cannot know.” (N2*,* G3).*

##### Sub-theme 3.2 a supportive ecosystem: academic mentorship and an empowering institutional culture

Participants described academic and mentor support, role models, and institutional culture as important sources of support when they encountered gendered judgements. According to participants, having a supportive instructor and an egalitarian institutional approach could shape whether they felt stronger and more visible throughout their educational and clinical training. One participant expressed this as follows: *“During placements*,* there can be a sense among patients and relatives that ‘men are taken more seriously’; in those moments*,* my instructor’s treating everyone equitably and supporting us made me feel that ‘what matters here is competence*,* not gender.’” (N4*,* G3).*

In addition, role models were described in terms of professional stance and authority. Students emphasised that, in mentorship, what mattered most was not the gender of academics or colleagues, but their ability to demonstrate strength and leadership. One participant noted that seeing strong and visible women in nursing in the clinic strengthened their professional commitment: *“When I see strong women in nursing in the clinic*,* I think*,* ‘I can stay here too.’” (N5*,* G6).*

Within the same sub-theme, participants also indicated that an institutional framework designed to discourage and address gender-based discrimination could provide an additional source of support. Such a framework was described as including written policies and procedures addressing gender-based discrimination and harassment; mechanisms, such as boards or commissions, for reporting concerns; practices intended to strengthen a culture of equity across educational and clinical processes; and awareness-raising training for both students and staff. In this regard, one participant stated: *“At our university*,* there is a committee directly conducting gender-related research; that is an important point in our institution. If I had a negative experience*,* knowing clearly where we can report it is reassuring.” (MD2*,* G4).* Another participant similarly noted: *“Every term*,* we are definitely given training on gender inequality. When the university supports this and conveys an ‘equality’ message through training*,* no one can continue gender inequality as if it were normal.” (M7*,* G2).*

##### Sub-theme 3.3 collective voice: professional solidarity and advocacy

Participants reported demonstrating professional solidarity in response to gender-based belittlement, labelling, and discriminatory discourse. They also described advocacy behaviours such as responding directly to gender-biased remarks, interrupting such discourse, or confronting the person involved. One participant illustrated this as follows:“It was a surgical rotation. *When the patient said*,* “I want a doctor who is a man*,*” my friend and I went into the room*,* and I gave a clear message: ‘The doctor who is a woman is highly competent in this area; when I work with her*,* I feel very safe.’ I didn’t want to leave my friend alone in that situation.” (MD4*,* G4).*

In contrast to these accounts, some participants noted that, in such situations, certain individuals might prefer to remain silent. One participant described a moment of gender-based belittlement in which a student who was a man did not intervene: *“During care*,* the patient and relatives assumed that my friend*,* who was a man studying nursing*, *was the doctor*,* and they approached me with a belittling and disrespectful attitude. While I was experiencing this*,* my friend remained silent. I think this shouldn’t be only a woman’s issue; we can transform society’s perceptions by supporting each other.” (N6*,* G6).*

## Discussion

This study examined how nursing, midwifery, and medical students interpreted gendered processes in educational and clinical learning environments in relation to professional identity, access to learning opportunities, psychological safety, and resilience. Findings were organised into three themes: gendered professional identity, inequity in clinical learning opportunities and psychological safety, and resilience through the lens of gender. Collectively, the findings suggest that gender-based discrimination may be reproduced not only through individual attitudes but also through participants’ experiences of clinical hierarchies, hidden curriculum processes, organisational culture, and power asymmetries [4, 10, 16].

A critical reading of the findings suggests that the clinical learning environment may function as more than a site of skill acquisition; it may also constitute a social and institutional space in which gendered authority, competence, visibility, and legitimacy are negotiated. Within the Turkish educational and clinical contexts represented in this study, students depended on educators, clinicians, mentors, physicians, patients, and patients’ relatives for supervision, assessment, professional acceptance, and access to practice. Such power asymmetries may have made perceived discriminatory practices more difficult to question or challenge. In this context, the hidden curriculum may serve as a process through which participants perceived patriarchal norms as being reproduced, even when these norms were not formally articulated as rules. Everyday practices, such as gendered task allocation, differential recognition, the normalisation of women’s care work as “expected,” questioning the credibility of physicians who are women, and positioning men in nursing as either doctors or as incongruent with care roles, may contribute to the continuation of patriarchal assumptions by making inequality appear natural, ordinary, or unavoidable.

The three student groups were analysed together because they shared clinical settings, worked within the same teams, and navigated similar hierarchical relationships and interactions with patients and patients’ relatives. Accordingly, the clinical environment was interpreted as a shared organisational context in which identity, competence, and status were negotiated alongside gender, supporting a holistic yet profession-sensitive comparative analytic lens [9, 16]. At the same time, participants’ accounts suggested that gendered hierarchies were not experienced identically across the three professions. In nursing, the visibility and recognition of students who identified as men appeared to be shaped by their minority position within a women-dominated profession. In midwifery, gendered expectations were closely associated with womanhood, motherhood, care, and bodily privacy and appeared to influence perceptions of who was considered legitimate in reproductive and maternal care [26]. In medicine, particularly in narratives concerning surgery, authority, technical competence, physical strength, and leadership were more strongly associated with masculinity. These profession-specific differences suggest that perceived glass-ceiling processes may operate through different mechanisms across health professions, depending on gender composition, occupational status, clinical authority, and culturally embedded assumptions about care, competence, and leadership [16, 27].

### Gendered professional identity: professional role, competence, and status

Participants’ accounts suggested that students’ professional identities were frequently interpreted through gendered expectations, role assumptions, and labels in both educational and clinical settings. Misused titles and blurred roles were described as contributing to experiences of misrecognition and influencing perceptions of respect, participation in team communication, willingness to assume responsibility, and professional legitimacy. Within the Turkish educational and clinical contexts represented in this study, this misrecognition appeared to be reinforced through everyday professional language. Expressions such as “male nurse,” as reported by participants, positioned men in nursing as exceptions to a feminised professional norm, while midwifery was closely associated with womanhood, motherhood, and reproductive care. This linguistic framing resonates with recent scholarship on gender-inclusive language in midwifery and perinatal services, which argues that professional terminology is closely connected to recognition, inclusion, justice, and professional representation [28, 29]. It also aligns with global evidence suggesting that midwives’ and nurse-midwives’ professional identity and public representation are closely related to social recognition, professional status, and the visibility of midwifery in the public realm [30]. From this perspective, nursing and midwifery students’ experiences of devaluation may be understood not only as local or interpersonal incidents but also as reflecting broader structural patterns in which feminised care professions remain less visible and less prioritised within health systems and their knowledge base may be treated as less authoritative [31].

This interpretation aligns with evidence that professions are co-coded through gender and prestige, with occupations culturally constructed as “feminine” often positioned as lower status [27], and with identity scholarship emphasising recognition and belonging as central to professional identity formation [4, 9, 13, 18]. Importantly, these processes also reflect power relations within clinical education. Assumptions such as “doctor = man” and “nurse/midwife = woman” do not merely mislabel students; they may also shape who is perceived as competent, authoritative, trustworthy, and suitable for leadership. In this sense, gendered professional labelling can operate as a subtle form of symbolic power that privileges masculinised authority while devaluing feminised care work. The repeated misrecognition of doctors who are women as nurses and men in nursing as doctors suggests that patriarchal assumptions may continue to shape professional status and clinical credibility.

A second key finding concerns the gendering of clinical labour through a “sensitive/fine work” versus “physical strength/crisis management” distinction. Participants described care and communication tasks as being channelled towards women, while physically demanding, emergency, and authority-linked tasks were more often associated with men. This pattern is consistent with persistent stereotypes and the implicit learning of “task suitability” through gender norms [7, 32, 33]. Such distinctions are not necessarily neutral; they may influence which competencies students can practise, how their competence is interpreted, and which future career pathways appear possible or legitimate [16].

The association of leadership, authority, and crisis management with masculinity suggests that perceptions of glass-ceiling barriers may emerge during training, before students formally enter the workforce. These findings are consistent with structural accounts of gender inequality in the health workforce [2, 3, 34] and suggest that hidden curriculum processes may contribute to students perceiving unequal career expectations as normal or inevitable. Midwifery students’ narratives are particularly important given the limited empirical literature on identity, status, and recognition during training; their accounts suggested that they also experienced gendered hierarchies and status-related constraints.

### Inequity in clinical learning opportunities and psychological safety

Participants described how gendered norms were perceived to shape access to clinical learning opportunities and psychological safety. Hostile or sexist climates, everyday sexism, unwanted physical contact, and sexist discourse have been associated in previous research with reluctance to speak up, reduced belonging, and weakened professional confidence [4, 32, 35]. In participants’ accounts, reduced psychological safety appeared to operate not only as an emotional response but also as a potential constraint on asking questions, seeking feedback, participating in care, and becoming visible within clinical teams.

For nursing and midwifery students, psychological unsafety was also linked to blurred public and clinical understandings of professional roles. The overlap and frequent confusion between nursing, midwifery, and nurse-midwifery identities may weaken students’ sense of professional distinctiveness, particularly in hierarchical clinical environments where recognition and authority are unevenly distributed. This interpretation is consistent with Pezaro et al. [30], who showed that midwives’ and nurse-midwives’ professional identity and public representation are closely connected to social recognition, professional status, and how midwifery is understood in the public realm. From this perspective, students’ micro-level experiences of exclusion, misrecognition, and reduced confidence may be situated within a broader macro-level context in which professional identity is shaped not only through clinical tasks but also through public perceptions and societal understandings.

Opportunity inequities were particularly evident in participants’ accounts of gendered access to clinical practice. Participants described women as being implicitly channelled away from some specialities, while nursing students who identified as men were excluded from obstetric, gynaecological, and urogenital procedures through instructions such as “don’t come” or “wait.” Although such practices were often justified in terms of privacy or patient preference, participants perceived them as creating unequal access to educational experiences and restricting opportunities for learning [7, 35]. Their comparison with intern physicians, who were described as encountering fewer constraints in similar contexts, suggests that these experiences may have reflected not only privacy concerns but also professional hierarchy and gendered status.

Inequity was also described through patterns of recognition and privilege. Students who identified as men, particularly in nursing and midwifery, were sometimes described as “rare” and therefore more visibly appreciated, whereas women’s work was treated as expected and rendered less visible. Such patterns may undermine psychological safety by affecting confidence, perceived value, and belonging [33]. They also suggest how the hidden curriculum may reward some identities while normalising the undervaluation of others. In this way, clinical education may contribute to the reproduction of patriarchal status arrangements not only by limiting access but also by shaping whose competence is noticed, praised, and legitimised.

Power asymmetries further appeared to shape students’ perceived capacity to challenge discrimination. Because students rely on educators, clinicians, mentors, patients, and patients’ relatives for assessment, access to learning, and professional acceptance, speaking up may carry perceived risks. Silence should therefore not be interpreted solely as passivity or individual reluctance; rather, in some contexts, it may represent a strategy for navigating perceived interpersonal or professional risks within hierarchical clinical cultures. At the same time, participants’ accounts of peer support and advocacy suggest that challenging gendered norms may require collective and institutional support in addition to individual action.

### Resilience through the lens of gender

This theme suggests that participants did not describe themselves as entirely passive in the face of gender-based discrimination; rather, they reported using individual and collective strategies to sustain their professional presence. Students linked visibility and acceptance to demonstrating competence, working harder, strengthening technical skills, and minimising errors, consistent with the role of self-efficacy in professional identity development [36]. However, these accounts also echo critiques that resilience discourse can shift responsibility for structural inequality onto individuals [37]. Although competence-building may support students’ confidence and professional identity, it does not in itself dismantle the structural conditions associated with gender-based discrimination. Indeed, the expectation that marginalised students must continually prove their competence, work harder, or avoid mistakes to be recognised as equal may inadvertently reinforce the very system they seek to challenge. Such dynamics may normalise a higher burden of proof for those whose legitimacy is already questioned within gendered clinical hierarchies.

Participants identified supportive academics, fair clinical educators, egalitarian institutional cultures, visible role models, and clear reporting pathways as protective conditions. Mentorship and organisational support were perceived as strengthening psychological safety and helping students resist discriminatory messages [11, 38]. In this context, written policies and reporting mechanisms may function as concrete safety infrastructures rather than merely symbolic measures. Participants’ accounts of solidarity and advocacy also point to the potential value of collective action. Speaking up against gender-biased remarks, supporting peers, and challenging gender-stereotypical assumptions may contribute to disrupting the normalisation of inequality. However, such actions may entail personal risk when institutional support is weak.

Therefore, the responsibility for challenging gender-based discrimination should not rest with students alone. Educational and clinical institutions should consider making power relations more visible, addressing hidden curriculum processes, protecting students who speak up, and developing accountable systems that support equitable learning. Overall, the findings suggest the importance of moving beyond an exclusively individualised understanding of resilience and attending to the educational and organisational conditions that may reproduce gendered inequality [39].

### Implications

The findings have important implications for health professions education and clinical training. Educational and clinical institutions may need to move beyond individual-level awareness initiatives and consider addressing the organisational conditions through which gender-based discrimination may be reproduced. This includes systematic attention to gendered role expectations, inequitable access to clinical practice, differential recognition of competence, hidden curriculum processes, and unsafe or unclear reporting pathways.

Clinical learning environments should aim to provide fair and transparent access to learning opportunities regardless of gender identity or professional programme. Supervisors, clinical educators, and mentors should be supported to recognise how everyday interactions, task allocation, feedback practices, and patient or relative preferences may reinforce power asymmetries and normalise gendered inequalities. In this regard, the hidden curriculum may warrant attention not only as an implicit educational process but also as a potential mechanism through which patriarchal norms are sustained when left unexamined.

Clear institutional policies, confidential reporting mechanisms, anti-discrimination procedures, supportive supervision, and visible gender-equitable role models may help foster learning environments in which students feel able to speak up without fear of negative academic or professional consequences. Although students’ competence-building, resilience, peer solidarity, and advocacy are valuable, sustainable change is likely to depend on institutional accountability and educational cultures that actively challenge gendered hierarchies, protect psychological safety, and support equitable professional identity development.

### Limitations

This study has several limitations. As a qualitative study conducted within a specific educational, clinical, and sociocultural context with a relatively small sample, the findings are not intended to be statistically generalisable, although they may offer insights transferable to comparable settings. Participants were recruited purposively through the researchers’ academic and professional channels and personal networks, which may have introduced self-selection bias and resulted in the inclusion of students who were more willing to discuss gender-related experiences. Data were generated through self-reported FGDs, and social desirability bias cannot be fully ruled out. Focus group dynamics may also have influenced disclosure, with some participants sharing more cautiously and dominant voices potentially shaping the discussion. The online format may have facilitated participation and accessibility but may also have limited observation of non-verbal interactions and affected the depth or spontaneity of group engagement.

The sample included substantially more participants who identified as women than as men, while only one participant identified as non-binary and one preferred not to disclose their gender identity. Consequently, the findings predominantly reflect the accounts of participants who identified as women and do not permit detailed interpretation of non-binary or other gender-diverse experiences. Although nursing, midwifery, and medical students were examined together because they shared clinical environments, the unequal representation of the three disciplines and the use of an integrated analysis may have limited the depth of profession-specific comparisons. The limited literature on midwifery students also constrained comparative interpretation. Furthermore, participants’ accounts included both personally experienced and witnessed incidents; therefore, the findings represent perceptions and interpretations of gendered experiences rather than independently verified events. Finally, the cross-sectional design precluded examination of how gender-related experiences and their perceived implications for professional identity development and psychological safety might change over time.

## Conclusion

This study suggests that gender-based discrimination may shape health professions students’ learning experiences, psychological safety, and professional identity formation across educational and clinical settings. The findings indicate that participants perceived discrimination as being reproduced not only through individual bias but also through clinical hierarchies, professional status differences, gendered task allocation, barriers to learning, and hidden curriculum processes. By making visible how gendered norms were perceived to be embedded in everyday clinical education, this study contributes to a more critical understanding of discrimination as a structural and relational phenomenon.

Participants described developing resilience through competence-building, mentorship, solidarity, and advocacy; however, these strategies should not be viewed as substitutes for institutional responsibility. Addressing gender-based discrimination may require clinical education systems to make power relations more visible, challenge normalised silence, promote equitable access to learning, and strengthen institutional accountability for safer and more inclusive health professions education. These conclusions should be interpreted within the specific educational, clinical, and sociocultural context of the study.

## Supplementary Information

Additional file 1: Consolidated criteria for reporting qualitative studies (COREQ): 32-item checklist.

## Data Availability

The data supporting this study’s findings are available on request from the corresponding author.
